# Gallstone ileus: What to do, when, and why: A case-based review of surgical options

**DOI:** 10.1016/j.ijscr.2025.111684

**Published:** 2025-07-18

**Authors:** Amr Elgazar, Mahmoud Diaa Hindawi, Ezzeldin Ahmed Abdelaty, Mohamed Ayman Salem, Abd-Elfattah Kalmoush, Ahmed K. Awad

**Affiliations:** a Department of General Surgery, Ain Shams University Hospitals, Cairo, Egypt; b Royal Free Hospital, Royal Free London Foundation Trust, Great London, United Kingdom of Great Britain and Northern Ireland; c Faculty of Medicine, Al-Azhar University, Cairo, Egypt; d Faculty of Medicine, Al-Azhar University, Dumyat, Al Jadidah, Egypt; e Faculty of Medicine, Damietta University, Dumyat, Al Jadidah, Egypt; f General Surgery Department, Al-Azhar University, Cairo, Egypt

**Keywords:** Case report, Gallstone ileus, Intestinal obstruction, Laparotomy, Enterolithotomy, Enterotomy

## Abstract

**Introduction:**

Gallstone ileus (GI) is a rare and serious complication of cholelithiasis, causing intestinal obstruction due to the migration of gallstones into the bowel lumen. It predominantly affects elderly patients and often lacks specific symptoms, delaying diagnosis and increasing mortality.

**Case presentation:**

We report a case of a 60-year-old male with signs of small bowel obstruction and a known history of gallstones. CT imaging confirmed a large gallstone impacted in the distal ileum. The patient underwent exploratory laparotomy with distal enterotomy and stone extraction, followed by an uneventful recovery.

**Discussion:**

This case illustrates the decision-making process in choosing between enterolithotomy, one-stage, and two-stage surgical approaches. A distal enterotomy was selected based on intraoperative anatomy, as the distal bowel was decompressed and healthier. Simultaneous cholecystectomy and fistula repair were deferred due to the patient's frailty, consistent with a two-stage strategy. A comprehensive literature review is provided to guide operative decision-making in similar cases.

**Conclusion:**

Anatomical and clinical context should guide surgical strategy in gallstone ileus. Distal enterotomy with enterolithotomy can be safe and effective in selected cases, with delayed biliary surgery reserved for symptomatic recurrence. Further research is needed to clarify the optimal timing and approach.

## Highlights

• Gallstone ileus is a rare cause of small bowel obstruction, typically occurring in elderly patients.
• CT showed a large obstructing gallstone in the distal ileum and a cholecysto-duodenal fistula.
• The patient underwent distal enterotomy only, avoiding bowel resection and biliary surgery.
• A one-stage procedure was avoided due to frailty; a safer two-stage approach was preferred.
• Surgical judgment is key; limited procedures like enterolithotomy may be safe and effective first steps.

## List of abbreviations

GI: Gallstone Ileus
ER: Emergency Department
CT: Computed Tomography
ICU: Intensive Care Unit
CRP: C-Reactive Protein
MRCP: Magnetic Resonance Cholangiopancreatography
PDS: Polydioxanone Suture
WBC: White Blood Cell
bpm: Beats Per Minute
mg/dL: Milligrams per Deciliter
g/dL: Grams per Deciliter

## 1 Introduction

Gallstone ileus (GI) is an emergency and rare complication of cholelithiasis where intestinal obstruction occurs due to the migration of gallstones to the intestine, often to the ileum through a cholecysto-enteric fistula, often connected to the duodenum [[1], [2], [3], [4]]. Among the cases of intestinal obstruction, GI represents 1 % to 4 % of all cases and up to 25 % of elderly cases, and among cholelithiasis patients, it represents 0.4 % to 1.5 % [5,6]. The most affected groups by GI are the elderly and women [7]. There are no specific symptoms for GI, which delays its diagnosis, making its mortality rate very high (15 %–30 %), also there are different surgical procedures for the management of GI (Enterolithotomy, one-stage surgery, and two-stage surgery) [5,8]. We present a case report of a large gallstone that was found impacted in the distal ileum and was removed by distal enterotomy. This paper has been reported in line with the SCARE criteria [9].

## 2 Case presentation

A 60-year-old male patient presented to our Emergency Department (ER) with abdominal pain, distension, and vomiting for three days, along with absolute constipation for 24 h. The patient had been in his usual state of health until three days ago, when he developed progressive colicky abdominal pain, mainly in the central abdomen. Over the past two days, he began experiencing bilious vomiting, which did not relieve his symptoms. In the last 24 h, he developed absolute constipation, with no passage of stool or flatus. He denied any history of weight loss, fever, or previous similar episodes.

The patient had a known history of gallstones diagnosed two years ago, with intermittent right upper quadrant pain, but he never sought treatment. He had also been diagnosed with type 2 diabetes mellitus 10 years ago, which was controlled with oral hypoglycemic drugs, and hypertension for 15 years, managed with Amlodipine 5 mg daily. He had no history of previous abdominal surgeries, was a non-smoker, and had no family history of malignancy.

On examination, the patient appeared stable. His vital signs were as follows: blood pressure of 130/80 mmHg, heart rate of 98 bpm, temperature of 37.2 °C, respiratory rate of 18 breaths per minute, and oxygen saturation of 98 % on room air. On abdominal examination, there was generalized abdominal distension with mild tenderness in the periumbilical and right lower quadrant. No signs of peritonitis were noted. On auscultation, high-pitched bowel sounds were present, suggesting bowel obstruction. Per rectal examination revealed an empty rectum, with no palpable masses or blood.

**Fig. 1:**
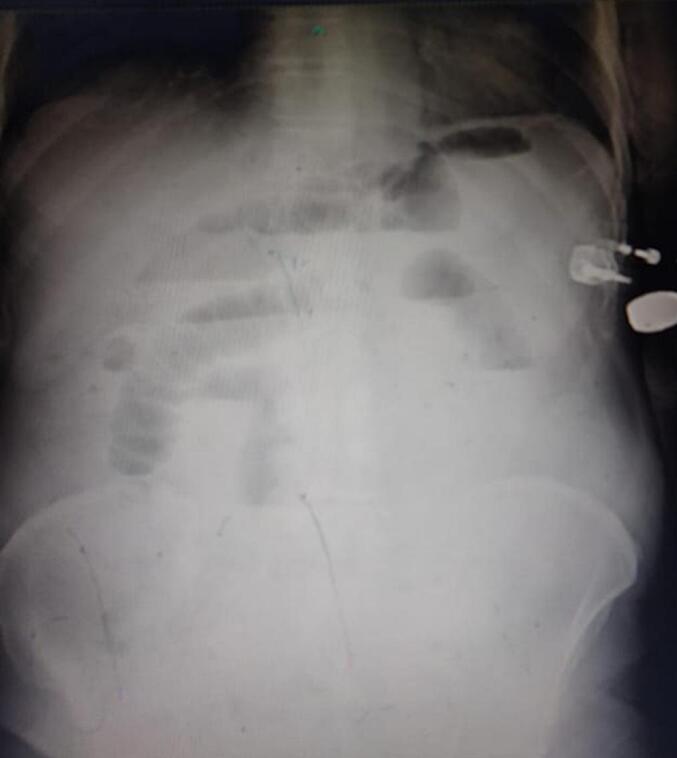
Erect abdominal x-ray showing multiple Air-Fluid levels.

**Fig. 2:**
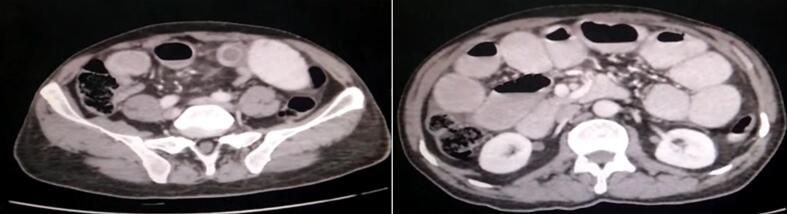
Axial CT abdomen with contrast showing a large stone impacted in the distal ileum with proximal small bowel dilatation and distal collapse.

**Fig. 3:**
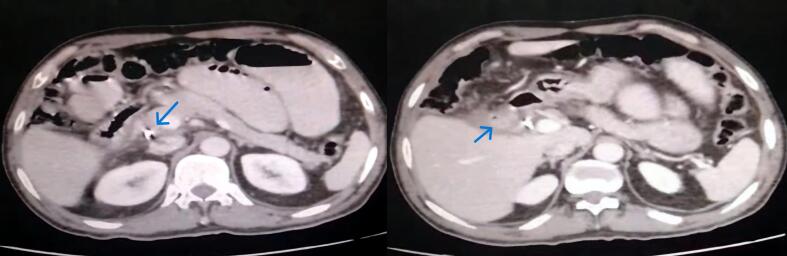
Axial contrast-enhanced CT image demonstrating a cholecysto-duodenal fistula. The image shows direct communication between the gallbladder and the adjacent duodenum (arrow), evidenced by pneumobilia and an indistinct gallbladder wall.

Laboratory investigations showed mild leukocytosis, with a white blood cell count of 12.5 × 10^9^/L. Hemoglobin was 13 g/dL, and C-reactive protein (CRP) was elevated at 40 mg/dL. Abdominal X-ray (erect and supine) revealed multiple air-fluid levels (Fig. 1). A CT abdomen with contrast confirmed the presence of dilated small bowel loops, with a large gallstone impacted in the distal ileum (Fig. 2). These findings suggested gallstone ileus, which is most likely caused by a cholecysto-duodenal fistula Fig. 3.

**Fig. 4:**
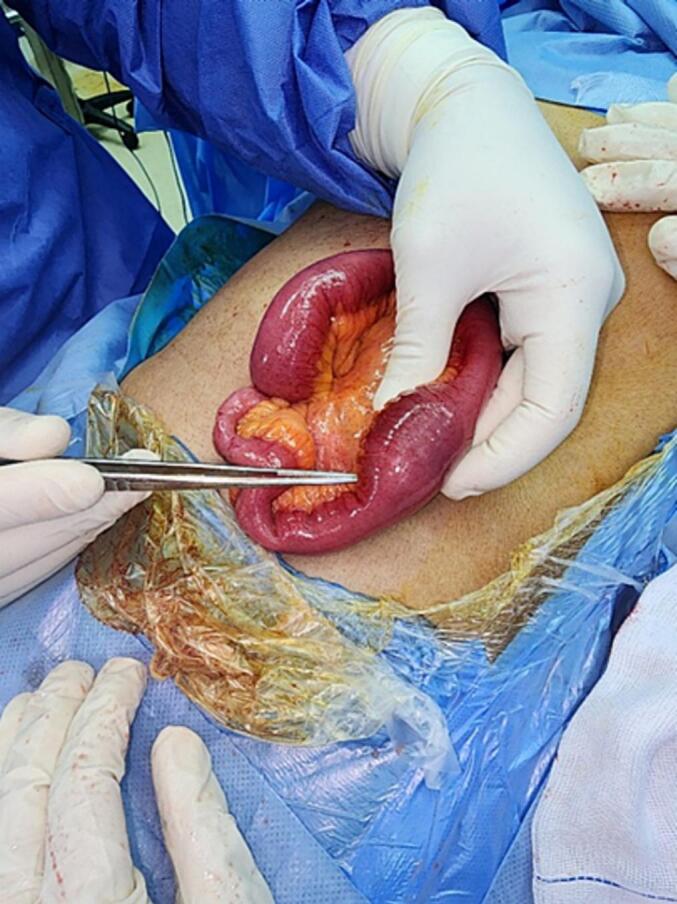
Large Gallbladder stone impacted in the distal ileum.

**Fig. 5:**
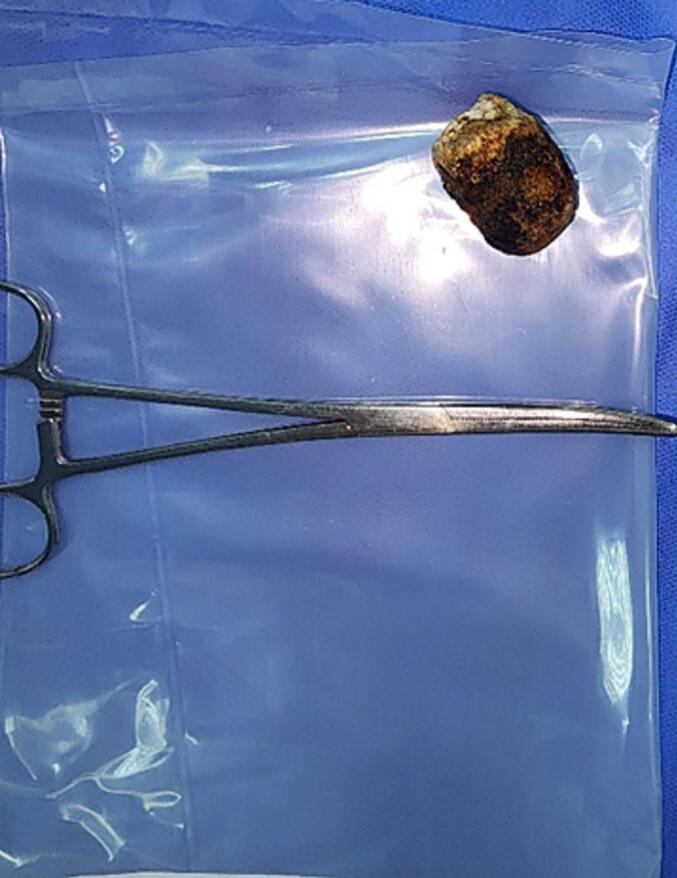
Stone after extraction.

The patient was resuscitated with intravenous fluids (Ringer's lactate) to correct dehydration and was admitted to the ICU preoperatively. He subsequently underwent an exploratory midline laparotomy, where a large gallstone was found impacted in the distal ileum. A distal enterotomy was performed, and the stone was extracted Fig. 4, Fig. 5. The enterotomy was then closed transversely using PDS 3/0 suture.

Postoperatively, the patient remained nil by mouth for two days. Free fluids were introduced on the third postoperative day, and he was discharged on the fifth day with an uneventful recovery.

## 3 Discussion

The first to describe the GI was Dr. Erasmus Bartholin, a Danish physician and mathematician [10]. GI has many risk factors, as it commonly occurs in women and the elderly (over 60 years old), cholelithiasis history with large stones (larger than 2 cm), episodes of acute cholecystitis, and Caucasians [8]. Moreover, GI diagnosis is difficult and requires laparotomy in 50 % of cases. However, 50 % can be diagnosed by plain abdominal films. The classic findings are: pneumobilia; intestinal obstruction, aberrant gallstone location; and change in the location of a previously observed stone [11].

Our patient's characteristics were commonly following the pathology of GI as he was a 60-year-old male with a history of gallstones, diabetes, and hypertension who presented with three days of abdominal pain, distension, vomiting, and absolute constipation also on radiology as on CT imaging confirmed a large gallstone impacted in the distal ileum, likely due to a cholecysto-duodenal fistula as shown in Fig. 2.

The main management of GI is surgery, and it aims to relieve the obstruction by removing the gallstone. In our case, we opted for a distal enterotomy rather than a proximal approach due to technical and anatomical considerations observed intraoperatively. The bowel proximal to the obstructing gallstone was significantly distended and edematous, making manipulation difficult and posing a risk of iatrogenic perforation or tearing. In contrast, the bowel distal to the stone was decompressed, healthy, and easier to handle, offering a safer and more technically feasible option for enterotomy and stone extraction. By gently milking the stone into the distal segment, we avoided undue traction and facilitated a cleaner, tension-free closure of the enterotomy. This strategy minimizes the risk of bowel injury and promotes smoother postoperative recovery.

Regarding the decision not to perform simultaneous cholecystectomy and fistula repair, it was primarily guided by the patient's age, frailty, and clinical presentation with small bowel obstruction. A one-stage procedure combining enterolithotomy with biliary surgery carries increased operative time, higher contamination risk, and greater potential for complications, particularly in elderly patients with inflamed, friable tissues. Our conservative approach prioritized patient safety and focused on resolving the acute obstruction. Should the patient develop recurrent biliary symptoms in the future, elective definitive surgery can be considered when the patient is more clinically stable and optimized.

Preoperative stabilization is essential with special attention to electrolyte balance [1]. Surgery for GI is still without a standard technique [12,13]. The known techniques are enterolithotomy alone, a one-stage procedure, and a two-stage procedure.

Enterolithotomy is a procedure that involves removing a gallstone through an enterotomy without resolving the fistula or underlying gallbladder disease. It is the most commonly performed technique as it effectively treats the obstruction while avoiding the risks of more extensive and complex surgery [8].

One-stage surgery includes specific biliary procedures: cholecystectomy or cholecystostomy, along with fistula closure and enterolithotomy. Supporters of this approach argue that it significantly lowers the risk of recurrence and reduces the percentage of gallbladder carcinoma occurrence from 15 % to 1 % [8].

Two-stage surgery involves performing enterolithotomy first, followed by a delayed cholecystectomy and fistula repair. Although only about 10 % of patients who undergo enterolithotomy experience recurrent biliary symptoms, this approach is recommended for younger patients at risk of future biliary complications and those with retained gallstones prone to recurrent gallstone ileus. In this emergency setting, preoperative ERCP was not feasible due to the patient's clinical instability and the urgent need to relieve obstruction. Furthermore, ERCP plays a limited role when the diagnosis of gallstone ileus is already confirmed on CT and there is no evidence of cholangitis or retained common bile duct stones. Should biliary symptoms arise during follow-up, ERCP or delayed surgical intervention can be reconsidered. However, there is no consensus on the optimal timing between the two stages, with intervals ranging from 4 weeks to 6 months [8]. Furthermore, Reisner and Cohen conducted the most extensive analysis of GI cases, comparing mortality rates between enterolithotomy and one-stage surgery. Their findings indicated that enterolithotomy had a lower mortality rate (11.7 %) compared to one-stage surgery (16.9 %), leading them to conclude that enterolithotomy is the preferred technique [8].

Recent case reports show the spectrum of presentations and diagnostic challenges of GI and variants [14,15]. Becker et al. [15] described a 71-year-old male with chronic, radiographically visible gallstone migration from the jejunum to the ileum over a period of years, without symptoms or typical findings such as pneumobilia or fistula. The case underscored the role of serial imaging, and the diagnostic value of serial imaging, and highlighted that even long-standing enteric stones can lead to delayed obstruction [14]. Similarly, Alamoodi [15] described a distal jejunal GI in a patient without a history of gallstone disease or significant comorbidities. CT scan confirmed pneumobilia, as well as obstruction due to a 2 cm stone. The case was successfully managed by enterolithotomy, highlighting the role of CT and the potential need for surgical intervention in these atypical cases [15].

Ferhatoğlu et al. [16] conducted a comprehensive review of 152 Bouveret's syndrome cases, which is a proximal variant of gallstone ileus. Their analysis showed that endoscopic management had a low success rate (29 %), especially for stones >2.5 cm, and failed in most cases with 4 cm stones. Surgical extraction via gastrotomy or enterotomy proved more effective, particularly in elderly or comorbid patients. They also reported that classical Rigler's triad was seen in only 30–35 % of Bouveret cases, reinforcing the need for high suspicion, and only 50 % of cases were diagnosed preoperatively. Their data support a tailored surgical approach, with two-stage surgery favored in high-risk patients to reduce mortality [16]. These findings align with our management strategy and emphasize the need for individualized surgical planning in GI.

## 4 Implications for future research

Future research should clearly define the role of distal enterotomy in gallstone ileus, especially in cases where the bowel is viable and uncomplicated. Intraoperative decision-making would benefit from standardized criteria to determine when enterotomy alone is appropriate versus when to escalate to bowel resection. Comparative studies are needed to assess outcomes across various surgical strategies, including distal enterotomy, classical enterolithotomy, one-stage, and two-stage approaches, with a focus on safety, recurrence, and recovery. The potential for minimally invasive techniques, including laparoscopic and robotic enterotomy or cholecystectomy, should also be explored in both emergency and elective stages. These approaches may reduce postoperative complications and hospital stays, particularly in elderly patients or those with comorbidities. Additionally, the diagnostic value of advanced imaging modalities, such as MRCP and dual-phase CT, should be further studied to enhance the detection of fistulas and residual stones. Multicenter registries and prospective studies are essential to develop robust, evidence-based surgical algorithms tailored to patient risk profiles and anatomical findings.

## 5 Conclusion

Gallstone ileus remains a rare but serious surgical emergency, with variable presentations and no universally accepted management strategy. In our case, distal enterotomy provided a safe and effective solution for gallstone removal, avoiding unnecessary bowel resection and preserving intestinal continuity. This highlights the importance of intraoperative assessment in guiding conservative but definitive intervention. While enterolithotomy remains the most common technique, one-stage procedures offer the advantage of addressing the biliary source but carry a higher operative risk. Two-stage surgery, with delayed cholecystectomy and fistula repair, may be better suited for younger or high-risk patients. The emerging role of minimally invasive techniques in both acute and delayed settings presents additional opportunities to improve outcomes. Ultimately, surgical management should be individualized, balancing patient stability, anatomical complexity, and long-term risk. This case also reinforces the importance of timely decision-making under real-world constraints, where clinical priorities may preclude complete biliary evaluation or definitive treatment in the same session. Further research is needed to refine these choices and optimize care for this challenging condition.

## Author contribution

Amr Elgazar, Mahmoud Diaa Hindawi, Ezzeldin Ahmed Abdelaty, Mohamed Ayman Salem, Abd-Elfattah Kalmoush, and Ahmed K. Awad contributed equally to the preparation and writing of this case report. Amr Elgazar and Ahmed K. Awad were the primary surgeon and assistant surgeon, respectively, responsible for the operative management of the patient. Mahmoud Diaa Hindawi contributed as a clinical observer during the patient's management. Abd-Elfattah Kalmoush assisted with the literature review and led the revision process in response to reviewer comments.

## Informed consent

Informed consent was obtained from the study patient.

## Consent for publication

All authors' consents were obtained by the corresponding author for publication.

## Ethical approval

Ethical review and approval were not required for this case report in accordance with local institutional guidelines.

## Guarantor

Amr Elgazar, Mahmoud Diaa Hindawi and Ahmed K. Awad.

## Disclosure

None of the authors received payments or services from their institution or third parties.

## Funding

Not available.

## Declaration of competing interest

There are no conflicts of interest.

## Data Availability

No datasets were generated or analyzed during our study.

## Appendix A Supplementary data

Supplementary video Image 1
